# Prevalence of asthma-like symptoms with ageing

**DOI:** 10.1136/thoraxjnl-2016-209596

**Published:** 2017-10-03

**Authors:** Debbie Jarvis, Roger Newson, Christer Janson, Angelo Corsico, Joachim Heinrich, Josep M Anto, Michael J Abramson, Anne-Marie Kirsten, Jan Paul Zock, Roberto Bono, Pascal Demoly, Bénédicte Leynaert, Chantal Raherison, Isabelle Pin, Thorarinn Gislason, Rain Jogi, Vivi Schlunssen, Cecilie Svanes, John Watkins, Joost Weyler, Antonio Pereira-Vega, Isabel Urrutia, Jose A Gullón, Bertil Forsberg, Nicole Probst-Hensch, H Marike Boezen, Jesús Martinez-Moratalla Rovira, Simone Accordini, Roberto de Marco, Peter Burney

**Affiliations:** 1 Population Health and Occupational Disease, National Heart and Lung Institute, Imperial College, London, UK; 2 MRC-PHE Centre for Environment and Health, Imperial College London, London, UK; 3 Department of Primary Care and Public Health, Imperial College London, London, UK; 4 Department of Medical Sciences: Respiratory, Allergy and Sleep Research, Uppsala University, Uppsala, Sweden; 5 Division of Respiratory Diseases, IRCCS Policlinico San Matteo Foundation, Department of Internal Medicine and Therapeutics, University of Pavia, Pavia, Italy; 6 Institute and Outpatient Clinic for Occupational, Social and Environmental Medicine, Clinical Center, Ludwig Maximilian University, Comprehensive Pneumology Centre Munich, German Centre for Lung Research, Muenchen, Germany; 7 ISGlobal, Centre for Research in Environmental Epidemiology (CREAL), Barcelona, Spain; 8 Universitat Pompeu Fabra (UPF), Barcelona, Spain; 9 CIBER Epidemiología y Salud Pública (CIBERESP), Madrid, Spain; 10 Institut Municipal d’Investigació Mèdica (IMIM), Barcelona, Spain; 11 School of Public Health and Preventive Medicine, Monash University, Melbourne, Victoria, Australia; 12 Pulmonary Research Institute at Lung Clinic Grosshansdorf, Grosshansdorf, Germany; 13 Department of Public Health and Pediatrics, University of Turin, Turin, Italy; 14 University Hospital of Montpellier, Montpellier, France; 15 Sorbonne Universités, Paris, France; 16 Inserm UMR 1152-Equipe Epidémiologie, Université Paris Diderot, Paris, France; 17 Inserm-U1219 Bordeaux Population Health Research Center, Bordeaux University, Bordeaux, France; 18 INSERM, IAB, Team of Environmental Epidemiology applied to Reproduction and Respiratory Health, Grenoble, France; 19 Department of Pédiatrie, CHU de Grenoble Alpes, Grenoble, France; 20 Department of Respiratory Medicine and Sleep, Landspitali, The National University Hospital of Iceland, Reykjavik, Iceland; 21 Faculty of Medicine, University of Iceland, Reykjavik, Iceland; 22 Tartu University Hospital, Lung Clinic, Estonia, Europe; 23 Department of Public Health, Aarhus University, Aarhus, Denmark; 24 National Research Centre for the Working Environment, Copenhagen, Denmark; 25 Centre for International Health, University of Bergen, Bergen, Norway; 26 Department of Occupational Medicine, Haukeland University Hospital, Bergen, Norway; 27 Public Health Wales, Cardiff, Wales; 28 University of Cardiff, Cardiff, Wales; 29 Department of Epidemiology and Social Medicine (ESOC), Faculty of Medicine and Health Sciences, StatUA Statistics Centre, University of Antwerp, Antwerp, Belgium; 30 Respiratory and Allergy Clinical Unit, Universitary Hospitalary Complex, Huelva, Spain; 31 Department of Respiratory, Galdakao Hospital, Galdakao, Spain; 32 Servicio Neumología, Hospital Universitario San Agustín, Avilés, Spain; 33 Public Health and Clinical Medicine, Occupational and Environmental Medicine, University of Umea, Umea, Sweden; 34 Department Epidemiology and Public Health, Swiss Tropical and Public Health Institute, Basel, Switzerland; 35 Department of Epidemiology, University of Groningen, University Medical Center Groningen, Groningen, The Netherlands; 36 Servicio de Neumología, Complejo Hospitalario Universitario, Albacete, Spain; 37 Facultad de Medicina Albacete, University of Castilla-La Mancha, Ciudad Real, Spain; 38 Unit of Epidemiology and Medical Statistics, Department of Diagnostics and Public Health, University of Verona, Verona, Italy

**Keywords:** Asthma Epidemiology

## Abstract

**Background:**

Change in the prevalence of asthma-like symptoms in populations of ageing adults is likely to be influenced by smoking, asthma treatment and atopy.

**Methods:**

The European Community Respiratory Health Survey collected information on prevalent asthma-like symptoms from representative samples of adults aged 20–44 years (29 centres in 13 European countries and Australia) at baseline and 10 and 20 years later (n=7844). Net changes in symptom prevalence were determined using generalised estimating equations (accounting for non-response through inverse probability weighting), followed by meta-analysis of centre level estimates.

**Findings:**

Over 20 years the prevalence of ‘wheeze’ and ‘wheeze in the absence of a cold’ decreased (−2.4%, 95% CI −3.5 to −1.3%; −1.5%, 95% CI −2.4 to −0.6%, respectively) but the prevalence of asthma attacks, use of asthma medication and hay fever/nasal allergies increased (0.6%, 95% CI 0.1 to 1.11; 3.6%, 95% CI 3.0 to 4.2; 2.7%, 95% CI 1.7 to 3.7). Changes were similar in the first 10 years compared with the second 10 years, except for hay fever/nasal allergies (increase seen in the first 10 years only). Decreases in these wheeze-related symptoms were largely seen in the group who gave up smoking, and were seen in those who reported hay fever/nasal allergies at baseline.

**Interpretation:**

European adults born between 1946 and 1970 have, over the last 20 years, experienced less wheeze, although they were more likely to report asthma attacks, use of asthma medication and hay fever. Decrease in wheeze is largely attributable to smoking cessation, rather than improved treatment of asthma. It may also be influenced by reductions in atopy with ageing.

**Key messages**

**What is the key question?**

- Has the prevalence of asthma-like symptoms changed in populations of young adults as they have aged?

**What is the bottom line?**

- In young adults followed for 20 years, the prevalence of asthma, use of asthma medication and self-reported hay fever/allergic rhinitis has increased, although, largely related to smoking cessation, the prevalence of wheeze has fallen.

**Why read on?**

- This prospective survey which includes follow-up data on adults collected over the last 20 years provides unique European-wide information on the evolution of asthma-like symptoms in adults as they have aged.

## Background

There are few population-based longitudinal studies which have described the evolution of the burden of respiratory symptoms and disease in populations of adults as they age. The European Community Respiratory Health Survey II (ECRHS), a large multicentre, predominantly Western European, community-based study of younger adults reported that over a 10-year period (1992–2002) the prevalence of wheeze and wheeze-related symptoms fell among participants, while at the same time, the prevalence of asthma attacks and treatment for asthma increased.^1^ This pattern of change, which was seen in all participating countries, was hypothesised as due to increased labelling (more ‘asthma’) and increased treatment (less wheeze) in those with relatively mild disease, possibly in response to the widespread adoption of guidelines for asthma treatment during the 1990s.

At the time it seemed unlikely that the overall decrease in wheeze symptoms would be related to decreased atopy within the ageing populations, as there was a contemporaneous increase in the prevalence of self-reported nasal allergies, and little evidence of change in measured serum specific IgE in a representative subsample of responders.^2^ The observed changes in symptom burden may have occurred in response to cessation of smoking with ageing,^3–5^ driven by improved tobacco control during the 1990s, but at the time of publication information on smoking habits of the study group was still unavailable.

The ECRHS has now completed the 20-year follow-up of participants (ECRHS III). Here we describe changes in the prevalence of respiratory symptoms in these adults over the 20-year follow-up period and assess whether observed changes in symptom prevalence are likely to be related to changes in use of asthma medication, changes in a proxy marker for allergy (the presence of nasal allergies) or changes in smoking behaviour. We report our findings adhering to the Standards for Reporting of Observational Epidemiology.

## Methods

In 1990–1992, forty-eight participating centres (mainly, but not only, in Western Europe) each selected an area with a population of at least 150 000 people. At least 1500 men and 1500 women aged 20–44 years were selected at random from a population-based sampling frame, and sent a questionnaire asking about symptoms and attacks of asthma in the last 12 months, current use of asthma medication and presence of nasal allergies including hay fever. A random sample of responders to this survey was invited for further tests, including an extended interviewer administered questionnaire and lung function tests.^6^

In 2000–2002, twenty-nine (of the original 48) centres (14 countries) sent the identical brief questionnaire to all individuals who had taken part in the clinical assessment. The methods and the results^1 7–9^ have been presented in full elsewhere. In Melbourne the target sample answered identical questions through interviewer administered questionnaire, and in Spain (five centres) questions on hay fever/nasal allergies were administered separately by interviewer questionnaire.

From 2008 to 2013, these 29 centres (28 in Europe and 1 in Australia) sent the identical questionnaire to all responders to the postal survey in ECRHS II (Spanish centres; target population consisted of responders to clinical assessment ECRHS II). In centres where administrative records were available, Data Protection legislation permitted and ethical approval was granted, the questionnaire was also sent to those who were eligible for ECRHS II but had not taken part at the time of ECRHS II.

Information on smoking was collected during clinical assessment in ECRHS I and ECRHS II, and from the postal survey in ECRHS III. In 26 centres information on chronic winter cough and chronic winter phlegm was collected from the subsample of responders to the clinical assessments at each phase of the survey (chronic cough/phlegm for ‘most days for as much as three months each year’ in the winter).

The relevant questions in the postal survey are shown in online supplementary appendix 1.

10.1136/thoraxjnl-2016-209596.supp1 Supplementary file 1

**Figure 1:**
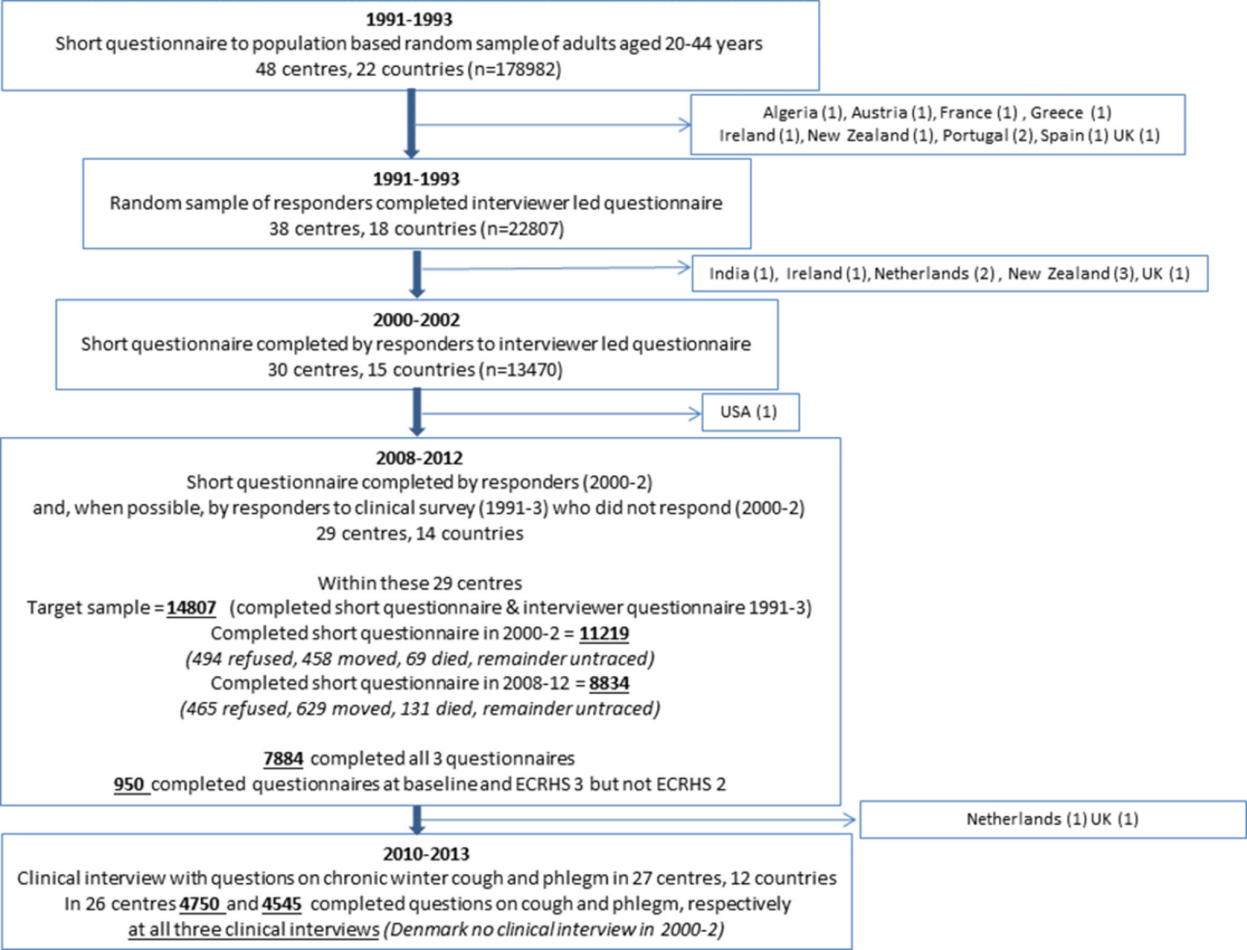
Study flow diagram for participants in the ECRHS III Canada (6) and Poland (1) not included—first data submitted after 1995. Note that ECRHS included follow-up of non-random sample of those selected because they had symptoms—this group is not included in this analysis. ECRHS, European Community Respiratory Health Survey.

The study flow is described in figure 1.

## Statistical methods

Absolute (‘net’) change in symptoms per 10 years of follow-up in those who responded to all three surveys was estimated using a logistic generalised estimating equations (GEE) model, with exchangeable correlation and Huber variances.^10^ As length of follow-up varied between centres, a quadratic reference spline was used^11^ to estimate the prevalence of symptoms in each population on 1 January 1991, 1 January 2001 and 1 January 2011. Analyses were conducted within centre and estimates combined using random effects meta-analysis^12^ and the I^2^ statistic^13^ used to assess heterogeneity between centres.

To adjust for non-response, analyses were conducted using inverse response-propensity weights^14^ in which ‘response’ was defined as ‘responded to all 3 surveys,’ and response odds determined multiplicatively (using separate logistic regressions in each centre) by age, gender, smoking status (never, ex, current) and reporting of wheeze in ECRHS I. Estimates from these adjusted analyses are presented.

Further analyses were stratified by gender (male or female), by age group at ECRHS I (<35 and 35+ years), by use of asthma treatments over the whole study period (never, ever), four smoking groups (non-smoker throughout period of follow-up, smokers at baseline who gave up during follow-up, smokers throughout follow-up and ex-smokers at follow-up with variable smoking history during the study) and presence of hay fever/nasal allergies at baseline.

Statistical analysis was carried out using Stata.^15^

## Results

### Response

Table 1 shows, by centre, the number of participants in the target sample (responders to the ECRHS I clinical survey), the number who responded to ECRHS II, and of these, the number who responded to ECRHS III. Overall, 53.2% (n=7884) of participants in the target sample completed all three questionnaires over the 20-year period, and 950 (6.42%) completed the questionnaire in ECRHS I and ECRHS III only. Response to all three questionnaires varied by centre (nine centres with response >60% and four centres less than 40%). The mean age at follow-up was 53.0 years with some variation in age at follow-up between centres, explained by a later start in ECRHS I in some centres (eg, Tartu, Estonia). Response to all three surveys was strongly related to being older, a female, a non-smoker and not having wheeze at baseline. Response rates were similar in those with and without diagnosed asthma (those reporting ‘asthma attack’ or ‘current asthma treatment’).

**Table 1:** Response rates for ECRHS II and III in the target sample (those responding to ECRHS I)

Country	Centre	Target sample*	Responded ECRHS II†		Responded ECRHS II and ECRHS III†				
		N	N	% of target sample	N	% of target sample	Mean follow-up (years)	% female	Mean age at ECRHS III
Iceland	Reykjavik	563	445	79.0	357	63.4	20.2	56.0	53.4
Norway	Bergen	835	658	78.8	505	60.5	19.0	49.5	50.9
Sweden	Umea	552	459	83.2	321	58.2	19.9	54.8	53.1
	Uppsala	622	513	82.5	387	62.2	19.9	53.0	52.7
	Gothenburg	682	548	80.4	414	60.7	19.9	56.0	52.6
Estonia	Tartu	431	352	81.7	229	53.1	18.2	58.5	49.4
Denmark	Aarhus	394	321	81.5	263	66.8	18.5	54.8	51.4
Belgium	Antwerp City	564	403	71.5	294	52.1	19.7	63.9	52.3
	Antwerp South	558	432	77.4	350	62.7	20.5	52.9	54.0
Germany	Hamburg	900	672	74.7	449	49.9	19.8	51.0	54.1
	Erfurt	715	548	76.6	453	63.4	18.9	50.3	52.6
Netherlands	Groningen	208	63	30.3	34	16.3	21.3	50.0	59.8
UK	Caerphilly	380	263	69.2	178	46.8	19.4	57.3	54.8
	Norwich	473	408	86.3	258	54.5	20.2	61.2	54.5
	Ipswich	448	389	86.8	234	52.2	19.9	56.4	54.3
Switzerland	Basel	852	513	60.2	306	35.9	19.0	50.7	52.0
France	Bordeaux	544	167	30.7	122	22.4	19.6	43.4	51.8
	Paris	651	493	75.7	388	59.6	20.3	55.4	55.7
	Grenoble	473	417	88.2	376	79.5	20.5	47.1	55.6
	Montpellier	456	285	62.5	177	38.8	19.3	53.1	56.1
Spain	Oviedo	357	310	86.8	175	49.0	18.7	51.4	53.1
	Galdakao	486	429	88.3	316	65.0	19.1	52.5	50.9
	Barcelona	392	314	80.1	205	52.3	18.8	59.5	51.5
	Albacete	435	393	90.3	223	51.3	19.5	54.7	51.8
	Huelva	271	223	82.3	133	49.1	18.9	58.6	51.5
Italy	Verona	342	254	74.3	150	43.9	16.3	47.3	49.9
	Pavia	310	288	92.9	156	50.3	20.1	50.6	55.0
	Turin	244	176	72.1	80	32.8	19.9	53.8	54.4
Australia	Melbourne	669	483	72.2	351	52.5	19.8	55.6	54.3
	Total	14 807	11 219	75.8	7884	53.2	19.5	53.8	53.0

*Target samples are responders to ECRHS I.

†Responders defined as subjects returning a questionnaire for ECRHS II or for ECRHS II and III.

ECRHS, European Community Respiratory Health Survey.

### Change in respiratory symptoms

Table 2 shows the absolute (or **‘**net’) change in respiratory symptoms adjusted for non-response during follow-up. The crude prevalences and non-response-adjusted prevalences at baseline are shown. It is the latter on which the change estimates are based. For most, but not all symptoms, adjusted estimates were marginally lower than the crude prevalence, reflecting the higher propensity for healthier subjects to respond to repeat postal surveys

**Table 2:** Baseline ECRHS I crude prevalences and ECRHS I inverse-propensity weighted prevalences with net change in prevalence of respiratory symptoms from ECRHS I to ECRHS II, from ECRHS II to ECRHS III, and from ECRHS I to ECRHS III

		ECRHS I subjects*		Number responding to ECRHS I, II and III							
Symptom from postal survey	Number of centres	Number of participants	ECRHS I crude prevalence (%)	Subjects	ECRHS I prevalence (%)	Net change ECRHS II-I (%)	Net change ECRHS III-II (%)	Net change ECRHS III-I (%) (95% CI)	p	Heterogeneity I^2^ (%)	Heterogeneity p
Wheeze	29	14 734	21.5	7738	21.3	−1.6	−0.6	**−2.4 (−3.5 to −1.3)**	**0.000015**	0.0	0.71
Wheeze with breathlessness	29	14 699	10.9	7630	10.1	−0.2	0.5	0.6 (−0.2 to 1.5)	0.12	0.0	0.61
Wheeze without a cold	29	14 720	13.4	7662	12.7	−0.5	−0.8	**−1.5 (−2.4 to −0.6)**	**0.0012**	0.0	0.84
Woken with chest tightness	23	14 365	14.3	6420	12.6	−0.5	−0.7	−1.3 (−3.0 to 0.3)	0.1	57.6	0.00033
Woken with shortness of breath	28	14 361	6.3	7459	5.5	0.3	0.4	0.9 (−0.1 to 2.0)	0.071	50.6	0.0013
Woken by attack of coughing	21	14 752	28.6	6279	27.8	−0.4	0.5	0.2 (−2.3 to 2.6)	0.91	65.0	0.000019
Asthma attack	29	14 745	3.9	7694	3.5	0.7	0.1	**0.6 (0.1 to 1.1)**	**0.013**	0.0	0.89
Current asthma medication	27	14 749	4.0	7069	3.5	2.0	1.5	**3.6 (3.0 to 4.2)**	**3.6×10^−31^**	0.0	0.56
Diagnosed asthma	27	14 711	5.5	7002	4.8	2.0	1.5	**3.5 (2.9 to 4.2)**	**2.6×10^−25^**	0.0	0.94
Nasal allergies	27	14 279	24.8	7020	23.2	2.8	−0.0	**2.7 (1.7 to 3.7)**	**1.2×10^−7^**	0.0	0.8
Symptom from interviewer survey on a subsample											
Chronic winter cough	26	14 698	7.7	4570	7.1	−0.3	1.4	1.1 (−0.3 to 2.5)	0.13	49.1	0.0027
Chronic winter phlegm	26	14 665	6.3	4545	5.8	0.4	0.6	1.0 (−0.3 to 2.2)	0.12	40.1	0.019

All estimates for change during the first 10  years of follow-up were compatible with estimates published in Chinn *et al* ^1^ (which were unadjusted for non-response, and included some centres which did not take part in ECRHS III).

*Crude prevalence in all target sample participants who completed postal survey in ECRHS I.

Numbers are in bold if p<0.05.

ECRHS, European Community Respiratory Health Survey.

Note that  changes in estimates from ECRHS I to ECRHS III are not the sum of changes from ECRHS I to ECRHS II, and from ECRHS II to ECRHS III due to the use of inverse variance weighting required by DerSimonian and Laird meta-analysis.^12^

**Figure 2:**
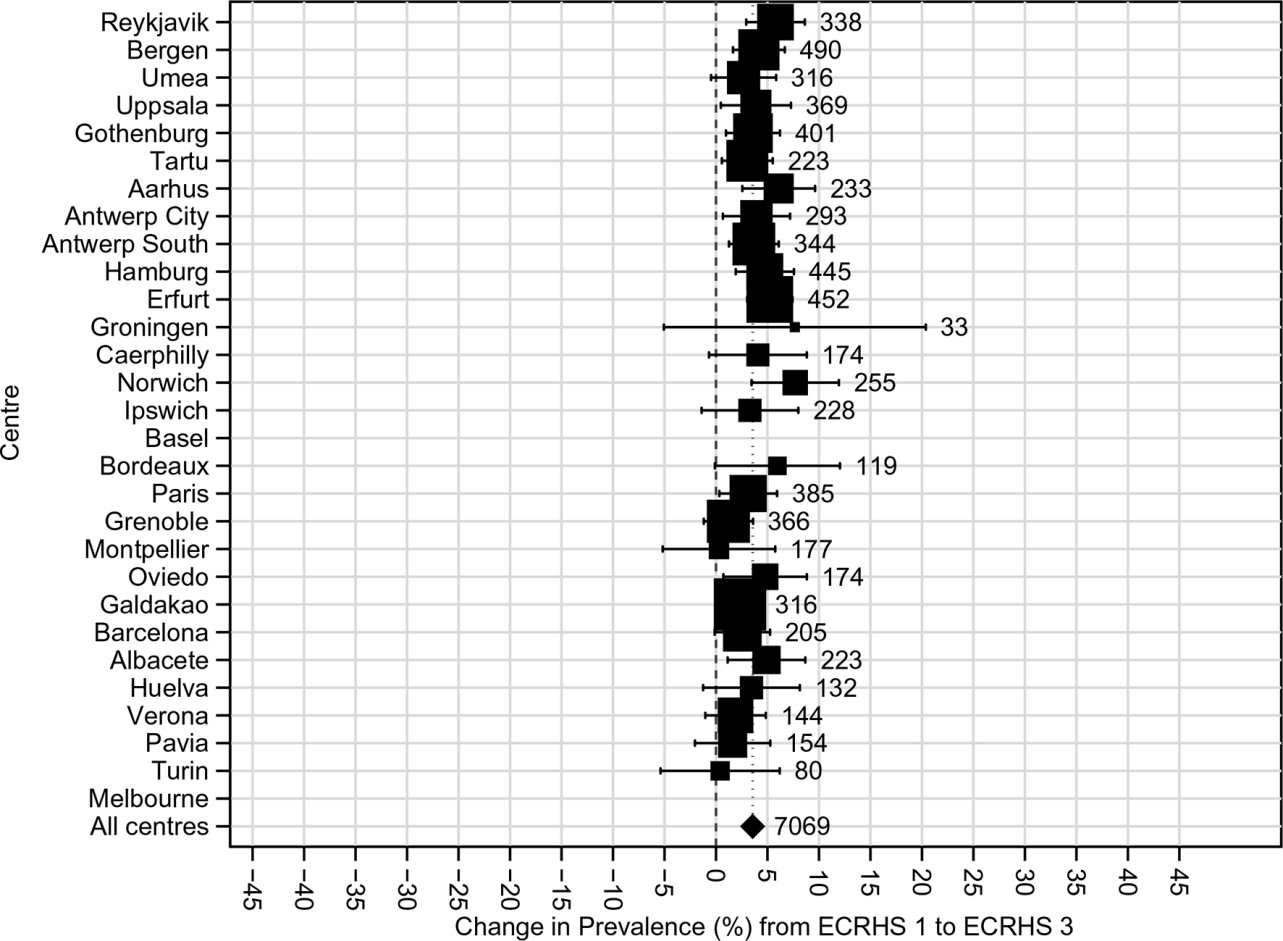
Net change in prevalence (%) of ‘current use of asthma medication’ (prevalence at ECRHS III minus prevalence at ECRHS I) adjusted for non-response and estimated by generalised estimating equations No estimate for Melbourne, Australia or Basel, Switzerland, as information on asthma medication use was not recorded during postal survey. ECRHS, European Community Respiratory Health Survey.

Over the 20-year period, the 12-month period prevalence of wheeze and wheeze in the absence of a cold fell (net change in prevalence −2.4%, 95% CI −3.5 to −1.3 and −1.5%, 95% CI −2.4 to −0.6%, respectively). There was no change in the prevalence of ‘wheeze with breathlessness,’ but there was a net increase in the prevalence of asthma attacks, current use of treatments for asthma (see figure 2) and current hay fever/nasal allergies. These observations were consistent across all centres (all p_heterogeneity_>0.5, all I^2^=0).

For the other asthma-like symptoms (waking with chest tightness, being woken by shortness of breath, being woken by coughing) the direction and magnitude of prevalence changes varied between centres (p_heterogeneity_<0.01, I^2^>50) with no obvious or consistent geographical pattern (see online supplementary figure E1a, E1b, E1c). Among the participants who completed the clinical survey at each of the three surveys and provided information on symptoms of chronic bronchitis, there were non-significant small increases in the prevalence of chronic winter cough and chronic winter phlegm with some variation between centres (p_heterogeneity_=0.0027 and 0.019, respectively; I^2^49.1% and 40.1%, respectively: see online supplementary figure E1d, E1e).

**Figure 3:**
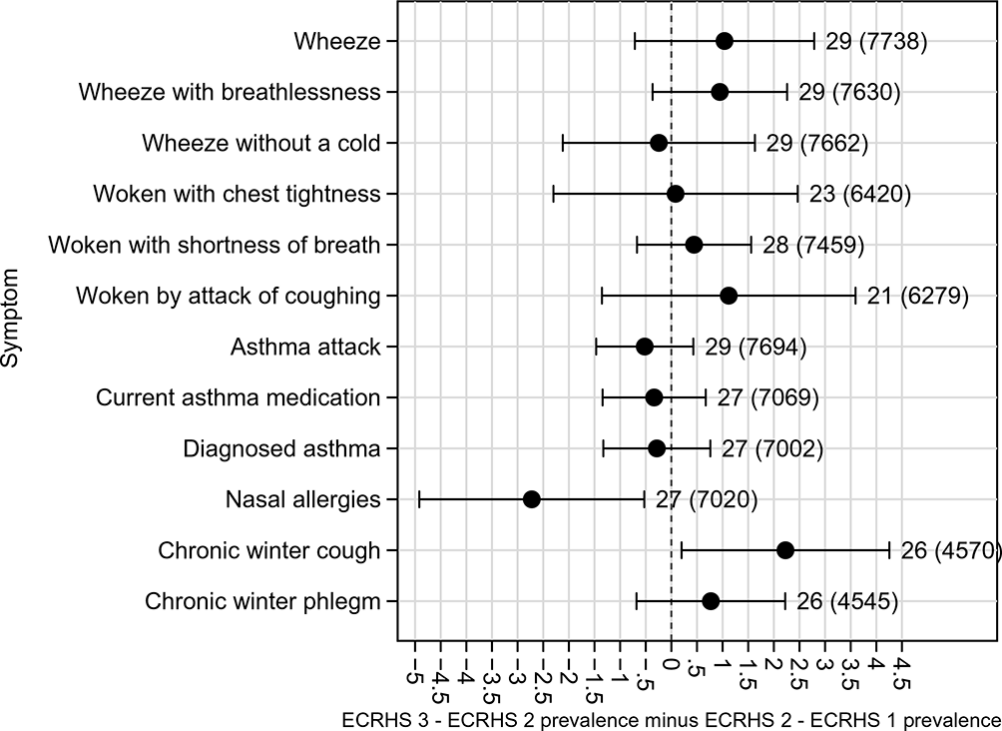
Comparison of change in prevalence in first 10 years to change in prevalence in second 10 years of follow-up (ie, ECRHS III-II change in prevalence minus ECRHS II-I change in prevalence) for all symptoms: adjusted for non-response and estimated by generalised estimating equations. ECRHS, European Community Respiratory Health Survey.

Changes in symptom prevalence in the first 10 years of follow-up were broadly similar to the changes that were seen in the second 10 years of follow-up (p>0.05, see figure 3), except for hay fever/nasal allergies (increases largely seen in the first, but not the second, 10 years of follow-up) and chronic winter cough (where the opposite was seen).

### Change in symptoms by age, gender, smoking history, treatment use and atopy

Table 3 shows net changes in the prevalence of wheeze, asthma attacks, use of asthma medication and hay fever/nasal allergies (ie, the symptoms that showed consistent patterns of change across all centres) in specific subgroups of the population.

**Table 3:** Net change in prevalence (%) of wheeze, asthma and nasal allergies/hay fever (prevalence at ECRHS III minus prevalence at ECRHS I); (adjusted for non-response) by generalised estimating equations and combined by random effects meta-analysis across countries and stratified by gender, age at baseline, presence of hay fever/nasal allergies at baseline, asthma treatment and smoking status

		Wheeze	Wheeze—no cold	Wheeze with shortness of breath	Asthma attack	Asthma medication	Hay fever/nasal allergies
Gender	Male	**−2.6 (−4.2 to −1.1)**	**−1.7 (**−**3.0 to −0.4)**	0.2 (**−**0.9 to 1.3)	0.2 (**−**0.5 to 0.9)	**2.5 (1.7–3.4)**	**2.3 (0.5–4.0)**
	Female	**−1.9 (**−**3.6 to −0.3)**	**−1.3 (**−**2.6 to −0.1)**	0.2 (**−**0.5 to 0.8)	0.0 (0.0–0.0)	**3.9 (2.8–5.0)**	**3.7 (2.0–5.4)**
Age group	<35	**−2.6 (**−**4.1 to **−**1.1)**	**−1.8 (**−**3.1 to −0.6)**	0.9 (**−**0.4 to 2.2)	**0.9 (0.2–1.5)**	**3.0 (2.2–3.8)**	**3.9 (2.5–5.3)**
(*Baseline*)	35+	**−1.9 (**−**3.5 to −0.4)**	−1.0 (**−**2.2 to 0.3)	−0.2 (**−**0.6 to 0.2)	0.2 (**−**0.5 to 0.9)	**3.9 (2.7–5.2)**	0.8 (**−**0.7 to 2.2)
Nasal allergies*	No	**−1.5** **(−2.9 to** **0.0)**	−0.3 (**−**1.6 to 1.1)	**1.8 (0.9–2.7)**	**1.2 (0.8 – 1.7)**	**3.7 (2.8–4.6)**	–
	Yes	****−**6.1** **(**−**9.4 to** ****−**2.9)**	****−**5.7 (**−**8.3 to **−**3.2)**	****−**3.7 (**−**6.7 to **−**0.7)**	**−**1.5 (**−**3.1 to 0.1)	**3.5 (1.6–5.4)**	**–**
Smoking*	Never	**−**0.1 (**−**2.2 to 1.9)	0.6 (**−**1.2 to 2.3)	0.4 (**−**0.9 to 1.8)	0.2 (**−**0.6 to 0.9)	**3.4 (2.1–4.7)**	2.1 (**−**0.3 to 4.5)
	Ex → Ex	5.3 (**−**4.1 to 14.7)	**−**0.1 (**−**2.2 to 2.0)	**−**0.6 (**−**2.6 to 1.5)	0.8 (**−**0.9 to 2.5)	**2.4 (1.1–3.7)**	**3.1 (0.6–5.6)**
	Current →Ex	****−**12.2 (**−**16.0 to **−**8.5)**	****−**7.4 (**−**11.4 to **−**3.3)**	0.6 (**−**1.8 to 3.0)	1.1 (**−**0.4 to 2.7)	**5.1 (3.5–6.7)**	**3.8 (0.4–7.2)**
	Current → Current	1.0 (**−**2.6 to 4.7)	0.0 (**−**3.7 to 3.7)	2.5 (**−**0.4 to 5.4)	3.3 (**−**0.8 to 7.4)	**4.6 (1.8–7.4)**	3.8 (**−**0.9 to 8.6)
Asthma medication	Never	**−**2.9 (**−**4.1 to 1.7)	**−**1.6 (**−**2.9 to **−**0.4)	0.3 (**−**0.6 to 1.2)	**−**0.0 (**−**0.3 to 0.3)	–	**2.7 (1.6–3.9)**
	Ever	0.8 (**−**3.9 to 5.5)	**−**1.4 (**−**7.3 to 4.5)	6.6 (**−**2.9 to 16.1)	**4.6 (0.2–9.0)**	–	1.5 (**−**2.5 to 5.6)

*Meta-analyses conducted by country to allow comparison of groups.

Numbers are in bold if p<0.05.

ECRHS, European Community Respiratory Health Survey.

Compared with men, women showed smaller decreases in wheeze symptoms and larger increases in use of asthma medication and hay fever/nasal allergies, although the 95% CIs overlapped (eg, net *decrease* in prevalence of wheeze in men −2.6%, 95% CI −4.2 to −1.1; in women −1.9%, 95% CI −3.6 to −0.3: net *increase* in prevalence of current use of asthma medication in men 2.5%, 95% CI 1.7 to 3.4; in women 3.9%, 95% CI 2.8 to 5.0). Changes were unrelated to age at the start of the study, except for hay fever/nasal allergies where net increases were greater in those who were younger at baseline (as has been noted at the 10-year follow-up^1^).

The pattern of change in prevalence of wheeze differed by the proxy marker for atopy (reporting of hay fever/nasal allergies at baseline), with greater decreases being seen in those with hay fever/nasal allergies. Net increases in the prevalence of asthma attacks occurred among those who did not have hay fever/nasal allergies at baseline (1.2%, 95% CI 0.8 to 1.7, p=1.6×10^−7^) and small net decreases, just below conventional levels of significance (−1.5%, 95% CI −3.1 to 0.1, p=0.066) occurred in those who did.

**Figure 4:**
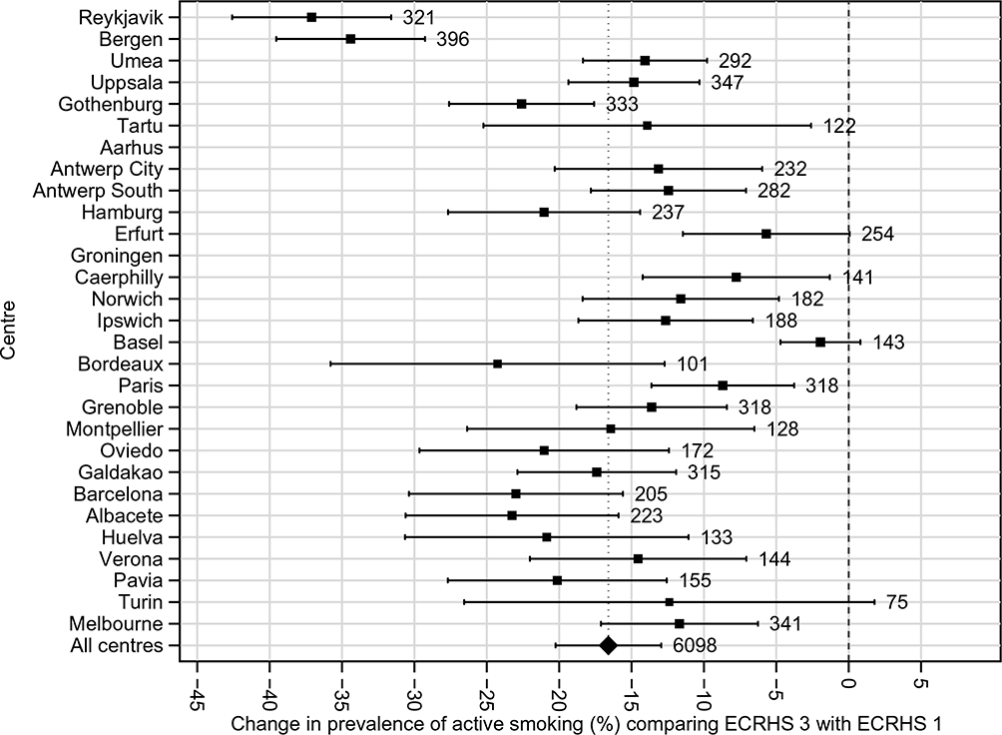
Net change in prevalence (%) of ‘current smoking’ (prevalence at ECRHS III minus prevalence at ECRHS I); adjusted for non-response and estimated by generalised estimating equations in each centre. ECRHS, European Community Respiratory Health Survey.

**Figure 5:**
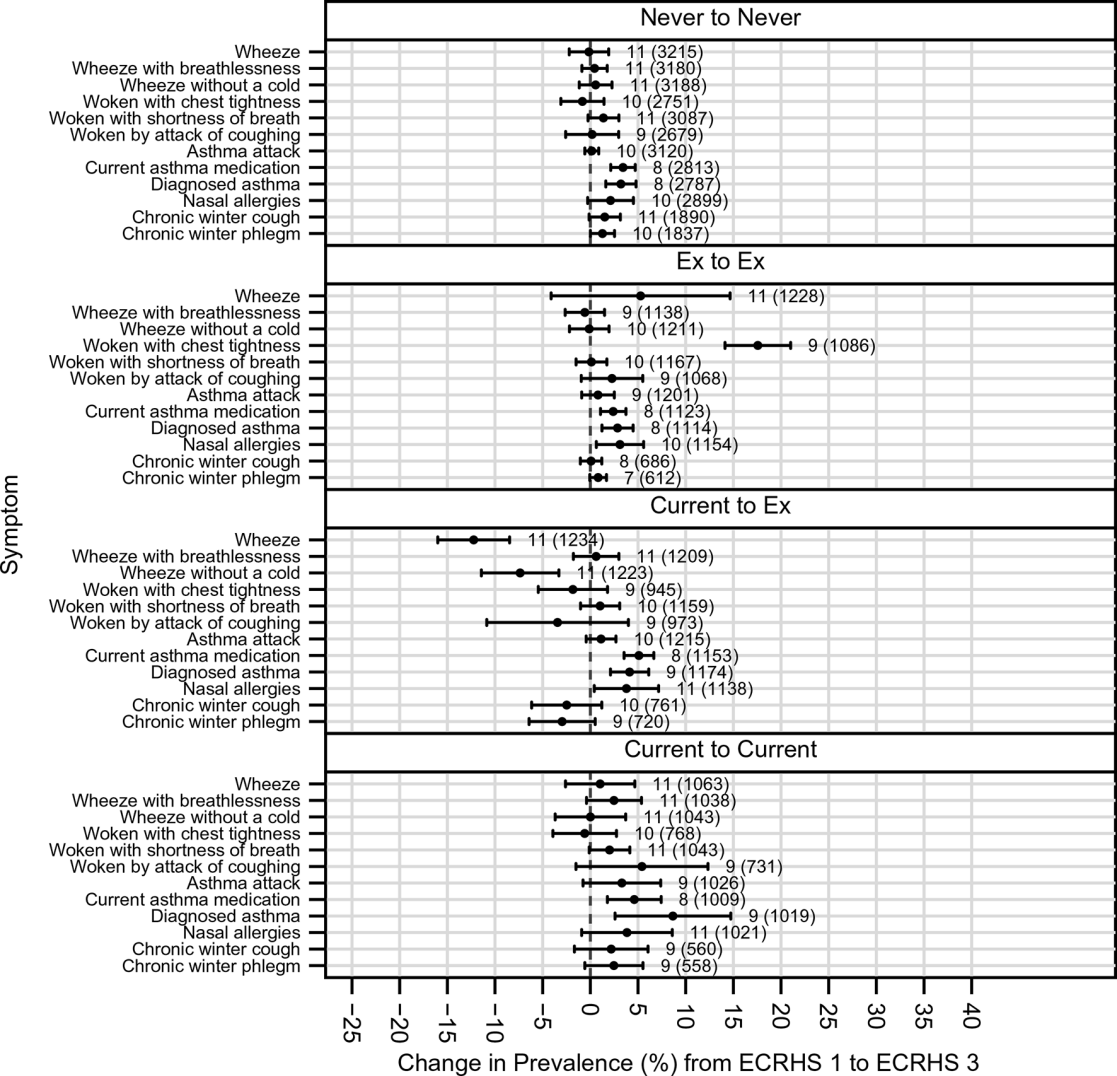
Net change in prevalence (%) of symptoms (prevalence at ECRHS III minus prevalence at ECRHS I); adjusted for non-response and estimated by generalised estimating equations stratified by smoking history. Some differences in numbers of countries for each analysis occur as information on smoking history in some centres differed. ECRHS, European Community Respiratory Health Survey.

During the first 10 years of follow-up the prevalence of smoking decreased (information collected during clinical interview^16^). It fell further during the second 10 years of follow-up with much larger decreases seen in some countries compared with others (net change in prevalence of active smoking over 20 years, −16.6%; 95% CI −20.3 to −12.9%; I^2^=89.9%; P_heterogeneity_=5.0×10^−40^; greatest change in Reykjavik, Iceland −37.1%, 95% CI −42.6 to −31.6%; least change in Basel, Switzerland −2.0%, 95% CI −4.7% to 0.8%) (figure 4). Larger net decreases in prevalence of wheeze, and wheeze in the absence of a cold, occurred among those who gave up smoking during the period of the study (figure 5), with a similar pattern of change for chronic winter cough and chronic winter phlegm. Changes in the prevalence of asthma attacks, use of asthma medication and of hay fever/nasal allergies were similar irrespective of smoking history.

**Figure 6:**
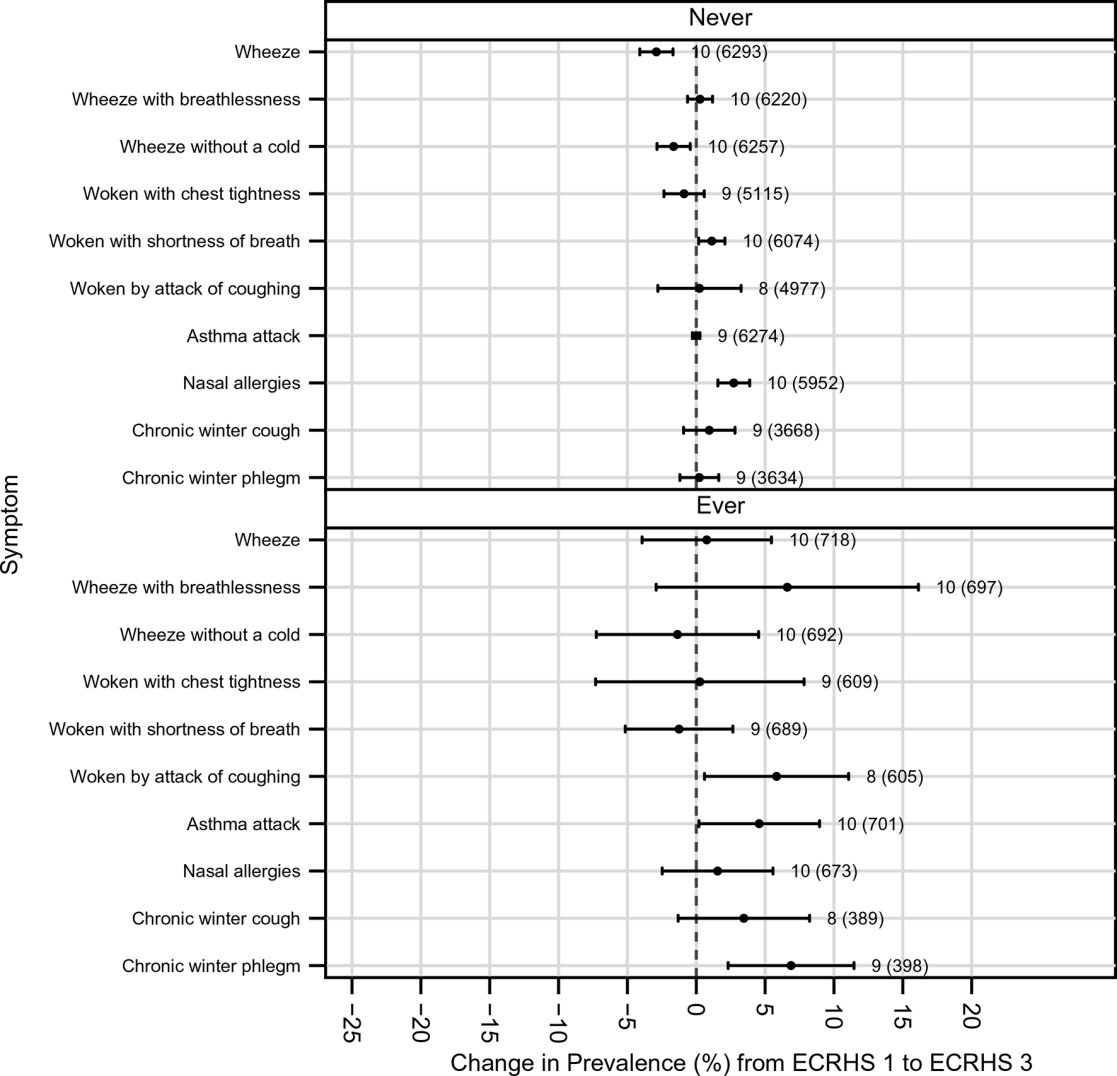
Net change in prevalence (%) of symptoms (prevalence at ECRHS III minus prevalence at ECRHS I); adjusted for non-response and estimated by generalised estimating equations stratified by use of asthma medication. ECRHS, European Community Respiratory Health Survey.

There was no evidence that those who received asthma treatment at some point during follow-up had greater decreases in the prevalence of wheeze than those who did not receive treatment. The net increase in asthma attacks (and in chronic winter cough and phlegm) occurred almost exclusively among those who had received asthma medication (shown in figure 6, which includes net changes in all symptoms).

## Discussion

Adults taking part in the ECRHS, an international multicentre cohort study, were less likely to report most non-specific respiratory symptoms (predominantly wheeze) as they aged over a period of 20 years, even though, in the same populations at the same time, there were marked increases in asthma, use of asthma medication and hay fever/nasal allergies. This decrease in wheeze symptoms at a group level, which was only seen in those who had never been treated for asthma, is most likely explained by the fall in active smoking as the cohorts have grown older. Changes in atopy may also have had an impact on the natural history of wheeze.

Information on the changing prevalence of symptoms in populations of adults as they age has been limited by the relative paucity of large cohort studies of adults with detailed repeat information on respiratory symptoms. The prevalence of wheeze, cough and phlegm increased in 900 Canadian adults aged 20–69 years followed for 10 years (1993–2003).^17^ Over a similar period, the prevalence of respiratory symptoms (wheeze, cough and asthma) increased in 1000 young (aged 16–34 years) English adults,^18^ and in Northern Italy the prevalence of asthma, allergic rhinitis and phlegm increased in about 800 participants followed for 25 years.^19^ In contrast, the Study of Health in Pomerania (SHIP)  study (in Germany) followed 3000 adults, aged 20–79 years, for about 6 years (1997/2001–2003/2006) and the prevalence of wheeze, waking with chest tightness and waking with shortness of breath fell (−0.7%, −1.9% and −1.0%, respectively) with some decrease in the prevalence of smoking (from 27.9% to 25.9%).^20^ Cohort studies such as SHIP^20^ and others in the UK,^21^ Norway^22^ and Chile^23^ show that change in the prevalence of respiratory symptoms is strongly determined by smoking uptake and smoking cessation patterns in the study populations.

In line with this, the most striking changes in respiratory symptom prevalence were seen in those who gave up smoking. Decreases in smoking within the cohort reflect changes in adult smoking rates that have occurred in Europe and elsewhere over the last years,^24^ but the link between decreases in smoking and decreases in symptoms has not always been demonstrated in studies using repeat cross-sectional data, rather than longitudinal data. Against a background of falling smoking rates, cross–sectional studies report the prevalence of respiratory symptoms to have decreased,^25–27^ been stable^28 29^ or increased.^30–32^

The ECRHS covers a longer time period (20 years) than most respiratory cohorts and has information from three follow-ups in populations with widely differing prevalences of respiratory symptoms, asthma and smoking cessation. There was a net increase in asthma attacks and use of asthma treatment (and hay fever/nasal allergies) over the last 20 years in all participating centres. This increase has occurred among those who have received treatment during the period of the study, reflecting both treatment in response to the development of asthma and possibly increasing severity of disease during the period of follow-up. From our data, there is no evidence that the population level net decrease in wheeze can be attributed to the more widespread use of asthma medication. The information on medication usage in this postal survey is too limited to determine whether people with asthma taking continuous regular daily medication (in particular inhaled steroids) throughout follow-up have become less symptomatic or showed slower disease progression compared with those who have taken little or irregular medication. Patients who adhere to regular treatment are likely to enjoy the best results but ‘regular treatment’ is not regular in most patients and maintenance treatment in real life should be assumed in most patients to be intermittent and symptom driven.^33^

Of interest we show that net decreases in wheeze symptoms are greater in those with hay fever/nasal allergies at baseline. In a representative subsample of ECRHS III responders there has been a decrease in serum-specific IgE sensitisation—particularly to house dust mite and cat.^34^ There was less evidence for decreases in sensitisation to grass, and, consistent with this, here we show substantial net increases in the prevalence of self-reported hay fever/nasal allergies. Possible interpretations of our observations include (1) some of those with symptoms of hay fever/nasal allergies at baseline have become less atopic with fewer wheeze symptoms and (2) those with hay fever/nasal allergies at baseline have received effective treatment of their nasal disorder leading to improvement in respiratory symptoms. Our findings are not contradictory with earlier observations from ECRHS^35^ showing that hay fever is a risk factor for new asthma onset, but may reflect a lower burden of wheeze symptoms in those with hay fever/nasal allergies as they grow older.

Further investigation of these observations is planned within the ECRHS sample with detailed clinical information, treatment information and serial measures of sensitisation (n~3500). Such analysis will be complicated by increases in the prevalence of hay fever/nasal allergies being greater in those born more recently. We observed this at the 10-year follow-up and it is consistent with the observation of cohort-related increases in the prevalence of serum-specific IgE to grass pollen within the ECRHS cohort.^34^ Furthermore, our observations need to be considered using all the information available from the clinical evaluations, and taking into account the full range of respiratory symptoms. In this report we have reported some data from these clinical interviews that demonstrate non-significant small increases in the prevalence of chronic winter cough and chronic winter phlegm of much lower magnitude than the increases in prevalence of asthma attacks or use of medication.

As with all cohorts, the ECRHS has some evidence of systematic loss to follow-up. We have used weighting procedures to account for non-response and our main observations are consistently seen across all centres irrespective of underlying response rates. It seems unlikely that systematic bias in loss to follow-up can account for the patterns we observed.

We have focused on changes in prevalence rather than present incidence and remission rates. The prevalence of disease is of direct relevance when assessing the burden of chronic disease in populations. Furthermore, incidence and remission rates can be biased by measurement error at both baseline and at follow-up, with an unknown proportion of study participants reporting symptoms when they do not have them (false positives) or denying symptoms when they do (false negatives). Even if relatively small, this measurement error can lead to erroneously high incidence and remission rate.^36^ Therefore, we have used GEE to derive changes in population prevalence of symptoms/disease as we have done in previous work^1^ and others have done when working with similar respiratory symptom data.^19 20 23^

Our report is using information derived from a postal survey on a large sample of adult and information on other relevant determinants of changes in the prevalence of respiratory symptoms (eg, body mass index) is not available. Analyses using information from detailed clinical assessment and incorporating other risk factors are in progress, but inevitably will be based on a smaller number of participants.

In conclusion, our study suggests that over the last 20 years, European adults born between 1946 and 1970, who have grown older over the last 20 years, have reduced their smoking and report fewer wheeze-related symptoms. Over the same period, the prevalence of asthma attacks, use of medication for asthma and hay fever/nasal allergies has risen. At a population level, the net decreases in symptoms are explained by smoking cessation. We cannot rule out that changes in atopy with ageing may have also contributed to these decreases.
