# Supplementary material for: Prevalence of asthma-like symptoms with ageing
Source: Thorax. 2017 Oct 3;73(1):37–48. doi: 10.1136/thoraxjnl-2016-209596 (PMC5738606; doi:10.1136/thoraxjnl-2016-209596)

List of funders
Financial support ECRHS I:

The co-ordination of ECRHS I was supported by the European Commission

The following grants helped to fund the local studies.

Australia: Asthma Foundation of Victoria, Allen and Hanbury's, **Belgium**: Belgian Science Policy Office, National Fund for Scientific Research, **Denmark: Aarhus** (R.Dahl, M.Iversen) **Estonia:** Estonian Science Foundation, grant no 1088, **France:** Ministère de la Santé, Glaxo France, Insitut Pneumologique d'Aquitaine, Contrat de Plan Etat-Région Languedoc-Rousillon, CNMATS, CNMRT (90MR/10, 91AF/6), Ministre delegué de la santé, RNSP, France; GSF, **Germany**: Bundesminister für Forschung und Technologie, **Greece:** The Greek Secretary General of Research and Technology, Fisons, Astra and Boehringer-Ingelheim; **Italy:** Ministero dell'Università e della Ricerca Scientifica e Tecnologica, CNR, Regione Veneto grant RSF n. 381/05.93, **Netherlands** Dutch Ministry of Wellbeing, Public Health and Culture and the Netherlands Asthma Foundation, **Norway:** Norwegian Research Council project no. 101422/310; **Portugal:** Glaxo Farmacêutica Lda, Sandoz Portugesa, **Spain:** Fondo de Investigación Sanitaria (#91/0016-060-05/E, 92/0319 and #93/0393), Hospital General de Albacete, Hospital General Juan Ramón Jiménez, Dirección Regional de Salud Pública (Consejería de Sanidad del Principado de Asturias), CIRIT (1997 SGR 00079) and Servicio Andaluz de Salud; **Sweden:** The Swedish Medical Research Council, the Swedish Heart Lung Foundation, the Swedish Association against Asthma and Allergy; **Switzerland:** Swiss national Science Foundation grant 4026-28099; **UK:** National Asthma Campaign, British Lung Foundation, Department of Health, South Thames Regional Health Authority**,**

ECRHS II

The co-ordination of ECRHS II was supported by the European Commission

The following grants helped to fund the local studies.

Australia: National Health and Medical Research Council**, Belgium:** Antwerp: Fund for Scientific Research (grant code, G.0402.00), University of Antwerp, Flemish Health Ministry**; Estonia: Tartu** Estonian Science Foundation grant no 4350, **France: (All)** Programme Hospitalier de Recherche Clinique—Direction de la Recherche Clinique (DRC) de Grenoble 2000 number 2610, Ministry of Health, Ministère de l'Emploi et de la Solidarité, Direction Génerale de la Santé, Centre Hospitalier Universitaire (CHU) de Grenoble, **Bordeaux**: Institut Pneumologique d'Aquitaine; **Grenoble:** Comite des Maladies Respiratoires de l'Isere **Montpellier:** Aventis (France), Direction Regionale des Affaires Sanitaires et Sociales Languedoc-Roussillon**; Paris:** Union Chimique Belge- Pharma (France),Aventis (France), Glaxo France, **Germany: Erfurt** GSF—National Research Centre for Environment and Health, Deutsche Forschungsgemeinschaft (grant code, FR1526/1-1) **Hamburg:** GSF—National

Research Centre for Environment and Health, Deutsche Forschungsgemeinschaft (grant code, MA 711/4-1) **Iceland: Reykjavik**, Icelandic Research Council, Icelandic University Hospital Fund; **Italy: Pavia** GlaxoSmithKline Italy, Italian Ministry of University and Scientific and Technological Research (MURST),Local University Funding for Research 1998 and 1999;**Turin**: Azienda Sanitaria Locale 4 Regione Piemonte (Italy), Azienda Ospedaliera Centro Traumatologico Ospedaliero/Centro Traumatologico Ortopedico—Istituto Clinico Ortopedico Regina Maria Adelaide Regione Piemonte **Verona:** Ministero dell'Universita´ e della Ricerca Scientifica (MURST), Glaxo Wellcome spa, **Norway: Bergen:** Norwegian Research Council, Norwegian Asthma and Allergy Association, Glaxo Wellcome AS, Norway Research Fund; **Spain:** Fondo de Investigacion Santarias (grant codes, 97/0035-01,99/0034-01 and 99/0034 02), HospitalUniversitario de Albacete, Consejeria de Sanidad; **Barcelona:** Sociedad Espanola de Neumologı´a y Cirugı´a Toracica, Public Health Service(grant code, R01 HL62633-01), Fondo de Investigaciones Santarias (grant codes, 97/0035-01, 99/0034-01, and 99/0034-02), Consell Interdepartamentalde Recerca i Innovacio´ Tecnolo`gica (grant code, 1999SGR 00241) Instituto de Salud Carlos III; Red deCentros de Epidemiologı´a y Salud Pu´blica, C03/09,Redde Basesmoleculares y fisiolo´gicas de lasEnfermedadesRespiratorias,C03/011and Red de Grupos Infancia y Medio Ambiente G03/176; **Huelva:** Fondo de Investigaciones Santarias (grant codes, 97/0035-01, 99/0034-01, and 99/0034-02); **Galdakao:** Basque Health Department **Oviedo:** Fondo de Investigaciones Sanitaria (97/0035-02, 97/0035, 99/0034-01, 99/0034-02, 99/0034-04, 99/0034-06, 99/350, 99/0034--07), European Commission (EU-PEAL PL01237), Generalitat de Catalunya (CIRIT 1999 SGR 00214), Hospital Universitario de Albacete, Sociedad Española de Neumología y Cirugía Torácica (SEPAR R01 HL62633-01) Red de Centros de Epidemiología y Salud Pública (C03/09), Red de Bases moleculares y fisiológicas de las Enfermedades Respiratorias (C03/011) and Red de Grupos Infancia y Medio Ambiente (G03/176);97/0035-01, 99/0034-01, and99/0034-02); **Sweden: Göteborg , Umea, Uppsala**: Swedish Heart Lung Foundation, Swedish Foundation for Health Care Sciences and Allergy Research, Swedish Asthma and Allergy Foundation, Swedish Cancer and Allergy Foundation, Swedish Council for Working Life and Social Research (FAS),**Switzerland: Basel** Swiss National Science Foundation, Swiss Federal Office for Education and Science, Swiss National Accident Insurance Fund; **UK: Ipswich and Norwich**: Asthma UK (formerly known as National Asthma Campaign)

Financial Support for ECRHS III

The co-ordination of ECRHS III was supported by the Medical Research Council (Grant Number 92091)

The following grants helped to fund the local studies.

Australia: National Health & Medical Research Council**, Belgium: Antwerp South, Antwerp City:** Research Foundation Flanders (FWO), grant code G.0.410.08.N.10 (both sites), **Estonia: Tartu-** SF0180060s09 from the Estonian Ministry of Education. **France:** (**All**) Ministère de la Santé. Programme Hospitalier de Recherche Clinique (PHRC) national 2010. **Bordeaux:** INSERM U897 Université Bordeaux segalen, **Grenoble:** Comite Scientifique AGIRadom 2011. **Paris:** Agence Nationale de la Santé, Région Ile de France, domaine d'intérêt majeur (DIM) **Germany : Erfurt:** German Research Foundation HE 3294/10-1 **Hamburg**: German Research Foundation MA 711/6-1, NO 262/7-1 **Iceland:** Reykjavik, The Landspitali University Hospital Research Fund, University of Iceland Research Fund, ResMed Foundation, California, USA, Orkuveita Reykjavikur (Geothermal plant), Vegagerðin (The Icelandic Road Administration (ICERA). **Italy:** All Italian centres were funded by the Italian Ministry of Health, Chiesi Farmaceutici SpA, in addition **Verona** was funded by Cariverona foundation, Education Ministry (MIUR). **Norway:** Norwegian Research council grant no 214123, Western Norway Regional Health Authorities grant no 911631, Bergen Medical Research Foundation. **Spain:** Fondo de Investigación Sanitaria (PS09/02457, PS09/00716 09/01511) PS09/02185 PS09/03190), Servicio Andaluz de Salud , Sociedad Española de Neumología y Cirurgía Torácica (SEPAR 1001/2010); **Sweden:** All centres were funded by The Swedish Heart and Lung Foundation, The Swedish Asthma and Allergy Association, The Swedish Association against Lung and Heart Disease.Fondo de Investigación Sanitaria (PS09/02457 **Barcelona:**Fondo de Investigación Sanitaria (FIS PS09/00716) Galdakao: Fondo de Investigación Sanitaria (FIS 09/01511) **Huelva**: Fondo de Investigación Sanitaria (FIS PS09/02185) and Servicio Andaluz de Salud **Oviedo:** Fondo de Investigación Sanitaria (FIS PS09/03190) **Sweden:** All centres were funded by The Swedish Heart and Lung Foundation, The Swedish Asthma and Allergy Association, The Swedish Association against Lung and Heart Disease. **Swedish Research Council for health, working life and welfare (FORTE) Göteborg :** Also received further funding from the Swedish Council for Working life and Social Research. Umea also received funding from Vasterbotten Country Council ALF grant. **Switzerland:** The Swiss National Science Foundation (grants no 33CSCO-134276/1, 33CSCO-108796, 3247BO-104283, 3247BO-104288, 3247BO-104284, 3247-065896, 3100-059302, 3200-052720, 3200-042532, 4026-028099) The Federal office for forest, environment and landscape, The Federal Office of Public Health, The Federal Office of Roads and Transport, the canton's government of Aargan, Basel-Stadt, Basel-Land, Geneva, Luzern, Ticino, Valais and Zürich, the Swiss Lung League, the canton's Lung League of Basel Stadt/ Basel, Landschaft, Geneva, Ticino, Valais and Zurich, SUVA, Freiwillige Akademische Gesellschaft, UBS Wealth Foundation, Talecris Biotherapeutics GmbH, Abbott Diagnostics, European Commission 018996 (GABRIEL), Wellcome Trust WT 084703MA, **UK:** Medical

Research Council (Grant Number 92091). Support also provided by the National Institute for Health Research through the Primary Care Research Network

Scientific teams

ECRHS I

Co-ordinating Centre (London): P Burney, S Chinn, C Luczynska†, D Jarvis, E Lai.
Project Management Group: P Burney (Project leader-UK) S Chinn (UK), C Luczynska † (UK), D Jarvis (UK), P Vermeire † (Antwerp), H Kesteloot (Leuven), J Bousquet (Montpellier), D Nowak (Hamburg), J Prichard † (Dublin), R de Marco† (Verona), B Rijcken (Groningen), JM Anto (Barcelona), J Sunyer (Barcelona), J Alves (Oporto), G Boman (Uppsala), N Nielsen (Copenhagen), P Paoletti (Pisa).
Participating Centres: **Australia: Melbourne** (M. Abramson, J. Kutin), **Belgium: Antwerp South, Antwerp Central** (P. Vermeire, F van Bastelaer) **Estonia: Tartu** (R. JÕgi), **France: Bordeaux** (A. Taytard) **Grenoble** (I. Pin, C. Pison), **Paris** (F. Neukirch, R.Liard) **Montpellier** (J. Bousquet, J. Knani), **Germany: Erfurt** (H-E Wichmann, J. Heinrich) **Hamburg** (H. Magnussen, D. Nowak), **Iceland: Reykjavik** (T. Gislason, D. Gislason) **Italy: Pavia** (A Marinoni, I .Cerveri) **Turin:** (M. Bugiani, C. Bucca, C. Romano) **Verona**: (R de Marco†, L. Cascio, C. Campello) **Netherlands: Groningen** (J. Droste, M.. Kerkhof) **Norway: Bergen** (A .Gulsvik, E. Omenaas) **Spain: Albacete** (J. Martinez-Moratalla, E. Almar, A.Mateos, M. Arévalo, A. Sánchez, M. Vizcaya, X. Aguilar, A. Teixidó) **Barcelona** (J.M. Antó, M. Kogevinas, J. Sunyer, F. Burgos, J. Castellsagué, J. Roca, JB. Soriano, A. Tobías) **Galdakao:** N. Muiñozguren , J. Ramos González, A. Capelastegui) **Huelva**: (J. Maldonado Pérez A Pereira, J. Sánchez) **Oviedo** (J. Quiros, I. Huerta, F. Payo) **Sweden: Göteborg** (N Lindholm, P Plaschke) **Uppsala**: (G Boman, C. Janson, E. Björnsson) **Umeå** (L. Rosenhall, E. Norrman B. Lundbäck) **Switzerland: Basel** (U Ackermann- Liebrich, N. Küenzli, A. Perruchoud) **UK: Caerphilly** (M. Burr†, J. Layzell) **Ipswich** (R. Hall) **Norwich** (B. Harrison)

ECRHS II

Co-ordinating Centre (London): P Burney, S Chinn, C Luczynska†, D Jarvis, J Knox.
Project Management Group: P Burney (Project leader-UK) S Chinn (UK), C Luczynska † (UK), D Jarvis (UK), P Vermeire † (Antwerp), J Bousquet (Montpellier), J Heinrich (Erfurt), M Wjst (Munich) R de Marco† (Verona), JM Anto (Barcelona), J Sunyer (Barcelona) C Janson (Uppsala), U. Ackermann-Liebrich (Basel), N. Kuenzli (University of Basel, and University of Southern California, Los Angeles, USA); F Neukirch (Paris)
Participating Centres: **Australia: Melbourne** (M. Abramson, E.H Walters, J. Raven), **Belgium: Antwerp South, Antwerp Central** (P. Vermeire, J. Weyler, M. van Sprundel, V. Nelen) **Estonia: Tartu** (R. JÕgi, A. Soon), **France: Bordeaux** (A. Taytard, C.Raherison),) **Grenoble** (I. Pin, J. Ferran-Quentin), **Paris** (F. Neukirch, B. Leynaert, R. Liard, M. Zureik) **Montpellier** (J. Bousquet, PJ Bousquet), **Germany: Erfurt** (J. Heinrich, M. Wjst, C. Frye, I. Meyer) **Hamburg** (H. Magnussen, D. Nowak), **Iceland: Reykjavik** (T. Gislason, E. Bjornsson, D. Gislason, K.B **Jörundsdóttir**) **Italy: Pavia** (A. Marinoni, S. Villani, M. Ponzio, F. Frigerio, M. Comelli, M. Grassi, I. Cerveri, A. Corsico) **Turin:** (R. Bono, M. Bugiani, P.Piccioni, E. Caria, A. Carosso, E. Migliore, G. Castiglioni) **Verona**: R. de Marco†, G. Verlato, E. Zanolin, S. Accordini, A. Poli, V. Lo Cascio, M. Ferrari, I. Cazzoletti) **Netherlands: Groningen** (M. Kerkhof) **Norway: Bergen** (A. Gulsvik, E. Omenaas, C. Svanes, B. Laerum) **Spain: Albacete** (J. Martinez-Moratalla Rovira, E. Almar, M. Arévalo, C. Boix, G González, J.M. Ignacio García, J. Solera, J Damián) **Barcelona** (J. M. Antó, J. Sunyer, M. Kogevinas, J. P. Zock, X. Basagaña, A. Jaen, F. Burgos, C. Acosta) **Galdakao:** (N. Muñozguren, J. Ramos, I. Urrutia, U. Aguirre) **Huelva**: (J. Maldonado, A. Pereira, J.L. Sanchez) **Oviedo** (F. Payo, I . Huerta, A. de la Vega, L Palenciano, J Azofra, A Cañada)

Sweden: Göteborg (K. Toren,L. Lillienberg, A. C. Olin, B. Balder, A. Pfeifer-Nilsson, R. Sundberg) **Uppsala**: (C. Janson, G. Boman, D. Norback, G. Wieslander, M. Gunnbjornsdottir) **Umeå** (E. Norrman, M. Soderberg, K.A Franklin, B. Lundback, B. Forsberg, L. Nystrom) **Switzerland: Basel** (N. Küenzli, B. Dibbert, M. Hazenkamp, M. Brutsche, U. Ackermann-Liebrich) **UK: Caerphilly** (M. Burr†, J. Layzell) **Ipswich** (D. Jarvis, R. Hall, D. Seaton) **Norwich** (D. Jarvis, B. Harrison)

ECRHS III

Co-ordinating Centre (London): D Jarvis, P Burney, M Tumilty, J Potts
Project Management Group: D Jarvis (UK), P Burney (UK), J Heinrich (Erfurt), R de Marco† (Verona), JM Anto (Barcelona) C Janson (Uppsala), K Toren (Goteburg), T Gislasson (Iceland) T Rochat (Basel), B Leyneart (Paris) C Svanes (Bergen) J Weyler (Antwerp) JP Zock (Barcelona)

Australia: Melbourne (M.Abramson, G. Benke, S. Dharmage, B. Thompson, S. Kaushik, M Matheson. **Belgium: South Antwerp & Antwerp City** (J. Weyler, H.Bentouhami, V. Nelen) **Estonia:** Tartu (R. JÕgi, H.Orru) **France: Bordeaux** (C. Raherison, P.O Girodet) **Grenoble** (I. Pin, V. Siroux, J.Ferran, J.L Cracowski) **Montpellier** (P. Demoly,A.Bourdin, I. Vachier) **Paris** (B. Leynaert, D. Soussan, D. Courbon, C. Neukirch, L. Alavoine, X. Duval, I. Poirier) **Germany: Erfurt** (J .Heinrich, E. Becker, G. Woelke, O. Manuwald) **Hamburg** (H. Magnussen, D. Nowak, A-M Kirsten), **Iceland: Reykjavik** (T. Gislason, B. Benediktsdottir, D. Gislason, E.S Arnardottir, M. Clausen, G. Gudmundsson, L. Gudmundsdottir, H. Palsdottir, K. Olafsdottir, S. Sigmundsdottir, K. Bara-Jörundsdottir), **Italy**: **Pavia** (I .Cerveri, A.Corsico, A. Grosso, F. Albicini, E. Gini, E.M Di Vincenzo, V. Ronzoni, S. Villani, F. Campanella, M. Gnesi, F. Manzoni, L. Rossi, O. Ferraro) **Turin:** (M. Bugiani, R. Bono, P. Piccioni, R. Tassinari, V. Bellisario, G. Trucco) **Verona:** (R de Marco†, S. Accordini, L. Calciano, L. Cazzoletti, M. Ferrari, A.M Fratta Pasini, F. Locatelli, P. Marchetti, A. Marcon, E. Montoli, G. Nguyen, M. Olivieri, C. Papadopoulou, C.Posenato, G. Pesce, P. Vallerio, G. Verlato, E. Zanolin), **Netherlands: Groningen** (H.M. Boezen), **Norway:** (C. Svanes, E. Omenaas, A. Johannessen, T. Skorge, F. Gomez Real) **Spain: Albacete** (J. Martinez-Moratalla Rovira, E. Almar, A. Mateos, S. García, A. Núñez, P.López, R. Sánchez, E Mancebo) **Barcelona:**(J-M. Antó, J.P Zock, J Garcia-Aymerich, M Kogevinas, X. Basagaña, A.E. Carsin, F. Burgos, C. Sanjuas, S Guerra, B. Jacquemin, P.Davdand **Galdakao**: N. Muñozguren, I. Urrutia, U. Aguirre, S. Pascual) **Huelva**:(J Antonio Maldonado, A. Pereira, J. Luis Sánchez, L. Palacios, **Oviedo**: (F. Payo, I. Huerta, N. Sánchez, M. Fernández, B. Robles) **Sweden: Göteborg** (K. Torén, M. Holm, J-L Kim, A-C. Olin, A. Dahlman-Höglund), **Umea** (B. Forsberg, L. Braback, L Modig, B Järvholm, H Bertilsson, K.A Franklin, C Wahlgreen) **Uppsala:**(B Andersson, D Norback, U Spetz Nystrom, G Wieslander, G.M Bodinaa Lund, K Nisser), **Switzerland: Basel** (N.M. Probst-Hensch, N. Künzli, D. Stolz, C. Schindler, T. Rochat, J.M. Gaspoz, E. Zemp Stutz, M.Adam, C. Autenrieth, I. Curjuric, J. Dratva, A. Di Pasquale, R. Ducret-Stich, E. Fischer, L. Grize, A. Hensel, D. Keidel, A. Kumar, M. Imboden, N. Maire, A. Mehta, H. Phuleria, M. Ragettli, M. Ritter, E. Schaffner, G.A Thun, A. Ineichen, T. Schikowski, M. Tarantino, M. Tsai, **UK: London** (P. Burney, D. Jarvis, S. Kapur, R. Newson, J. Potts,) **Ipswich:** (D Jarvis, M Tumilty, N. Innes) **Norwich**: (D Jarvis, M Tumilty, A. Wilson)

Questions from the ECRHS postal survey

1. Have you had wheezing or whistling in your chest at any time in the last 12 months?

    If NO – Go to question 2

    If YES

    1a Have you been at all breathless when the wheezing noise was present?

    1b Have you had this wheezing or whistling when you did not have a cold?

2. Have you woken up with a feeling of tightness in your chest at any time in the last 12 months?
3. Have you been woken by an attack of shortness of breath at any time in the last 12 months?
4. Have you been woken by an attack of coughing at any time in the last 12 months?
5. Have you had an attack of asthma in the last 12 months?
6. Are you currently taking any medicine (including inhalers, aerosols or tablets) for asthma?
7. Do you have any nasal allergies including hay fever?

TABLE E1. Baseline ECRHS I crude prevalences, unweighted ECRHS I prevalences and changes in prevalence of respiratory symptoms from ECRHS 1 to ECRHS2, from ECRHS 2 to ECRHS3 and ECRHS1 to ECRHS3

	*ECRHS I subjects: **		*ECRHS I-III subjects: †*										
Symptom	*Subjects*	*ECRHS I Crude Prev. (%)*	*Centres*	*Subjects*	*ECRHS I prev. (%)*	*Net change ECRHS II-I (^)*	*Net change ECRHS III-II (%)*	*Net change ECRHS III-I (%)*	*(95%*	*CI)*	*P*	*Het. I^2*	*Het P*
Wheeze	14734	21.5	29	7781	19.6	-1.0	-0.7	-1.7	(-2.8,	-0.7)	.00079	0.0	.88
Wheeze with breathlessness	14699	10.9	29	7683	9.5	-0.1	0.2	0.4	(-0.3,	1.2)	.25	0.0	.78
Wheeze without a cold	14720	13.4	29	7711	11.7	-0.2	-0.7	-1.2	(-2.0,	-0.4)	.0049	0.0	.83
Woken with chest tightness	14365	14.3	23	6465	12.5	-0.5	-0.8	-1.4	(-3.0,	0.1)	.071	56.0	.00058
Woken with shortness of breath	14361	6.3	28	7506	5.5	0.2	0.4	0.8	(-0.2,	1.8)	.1	48.8	.0022
Woken by attack of coughing	14752	28.6	21	6346	27.0	-0.1	0.5	0.1	(-2.0,	2.2)	.93	55.6	.0011
Asthma attack	14745	3.9	29	7767	3.5	0.7	0.1	0.6	(0.1,	1.1)	.012	0.0	.89
Current asthma medication	14749	4.0	27	7132	3.5	2.0	1.5	3.4	(2.8,	4.1)	3.2×10^{-26}	14.2	.26
Diagnosed asthma	14711	5.5	27	7063	4.8	2.1	1.5	3.4	(2.8,	4.1)	1.6×10^{-25}	0.0	.82
Nasal allergies	14279	24.8	27	7067	23.5	2.8	-0.1	2.6	(1.7,	3.6)	1.7×10^{-7}	0.0	.66

*Crude prevalence in all subjects assessed in ECRHS I

†Unweighted prevalences and their differences in all subjects assessed in ECRHS I to 3

NB all estimates for change during the first ten years of follow-up compatible with estimates published in Chinn et al 2004[1] (which were unadjusted for non-response, and included some centres which did not take part in ECRHS 3).

NB Changes in estimates from ECRHS I to ECRHS III are not the sum of changes ECRHS I to ECRHS II, and ECRHS II to ECRHS III due to the use of inverse variance weighting required by DerSImonian and Laird meta-analysis[12]

Figure E1 (a). ECRHS III - ECRHS I change in prevalence (adjusted for non-response) estimated by generalised estimating equations in each centre for waking with chest tightness (NB no data from SPAIN or Denmark as question not asked at ECRHS II)

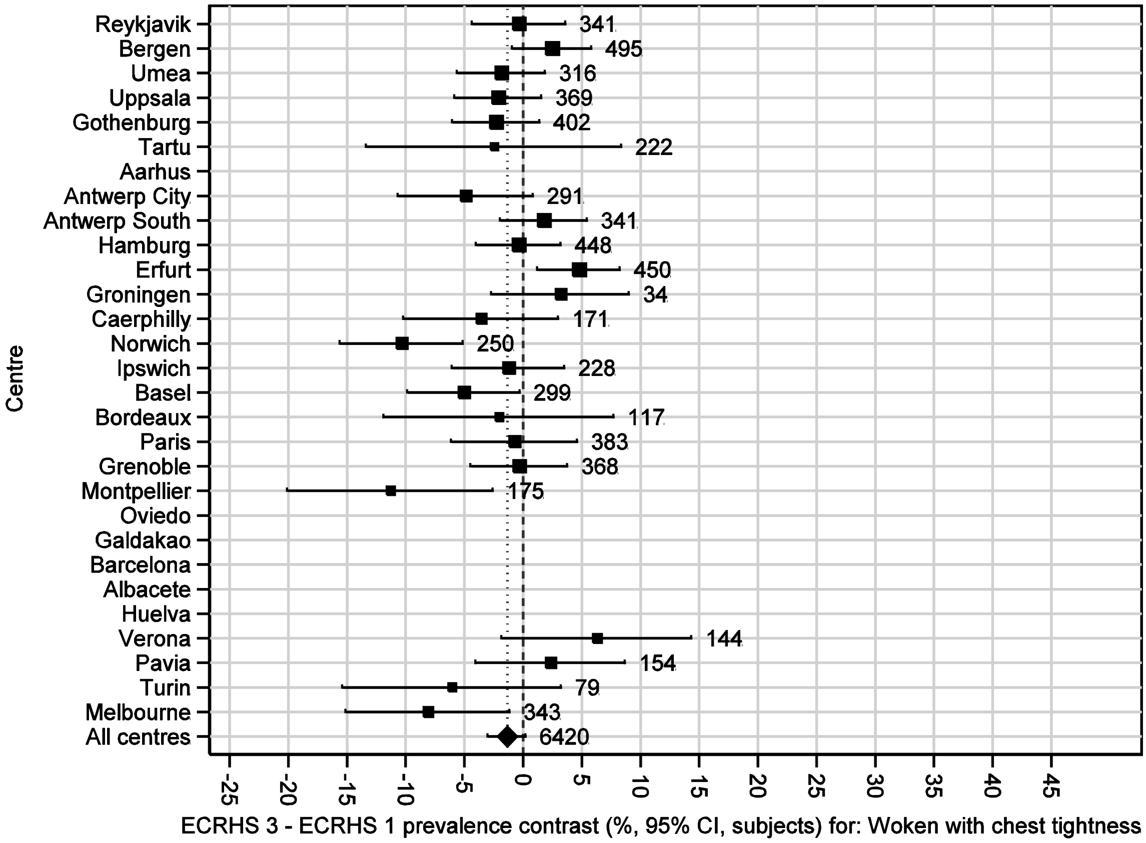

Figure E1(b). ECRHS III - ECRHS I change in prevalence (adjusted for non-response) estimated by generalised estimating equations in each centre for being woken with shortness of breath

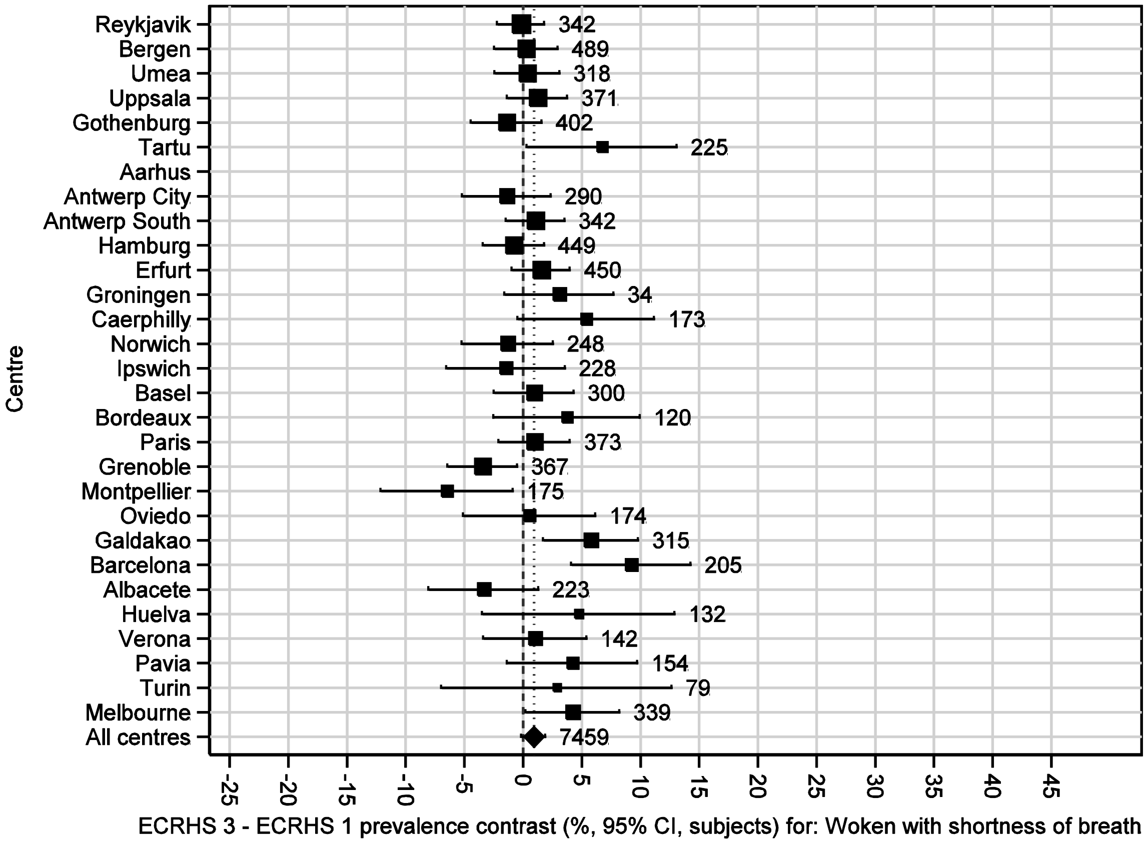

Figure E1(c). ECRHS III - ECRHS I change in prevalence (adjusted for non-response) estimated by generalised estimating equations in each centre for being woken with coughing

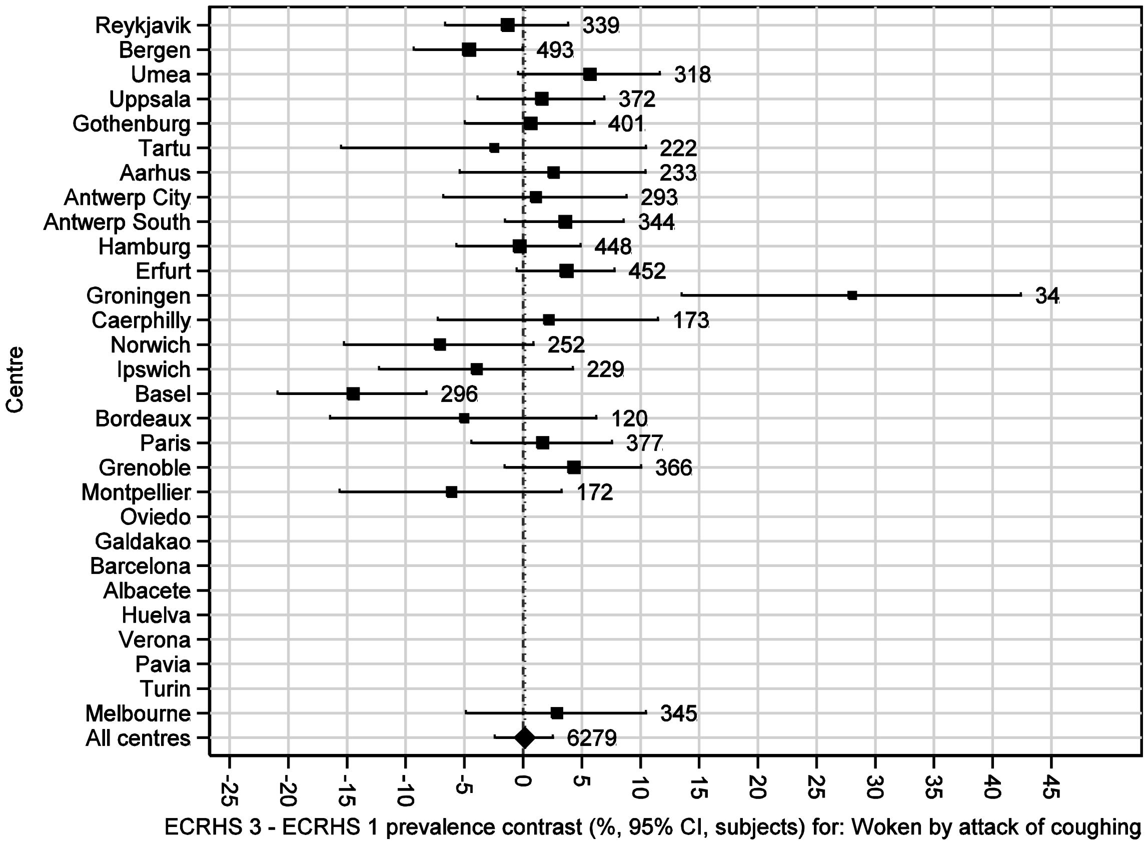

Figure E1(d). ECRHS III - ECRHS I change in prevalence (adjusted for non-response) estimated by generalised estimating equations in each centre for chronic winter cough

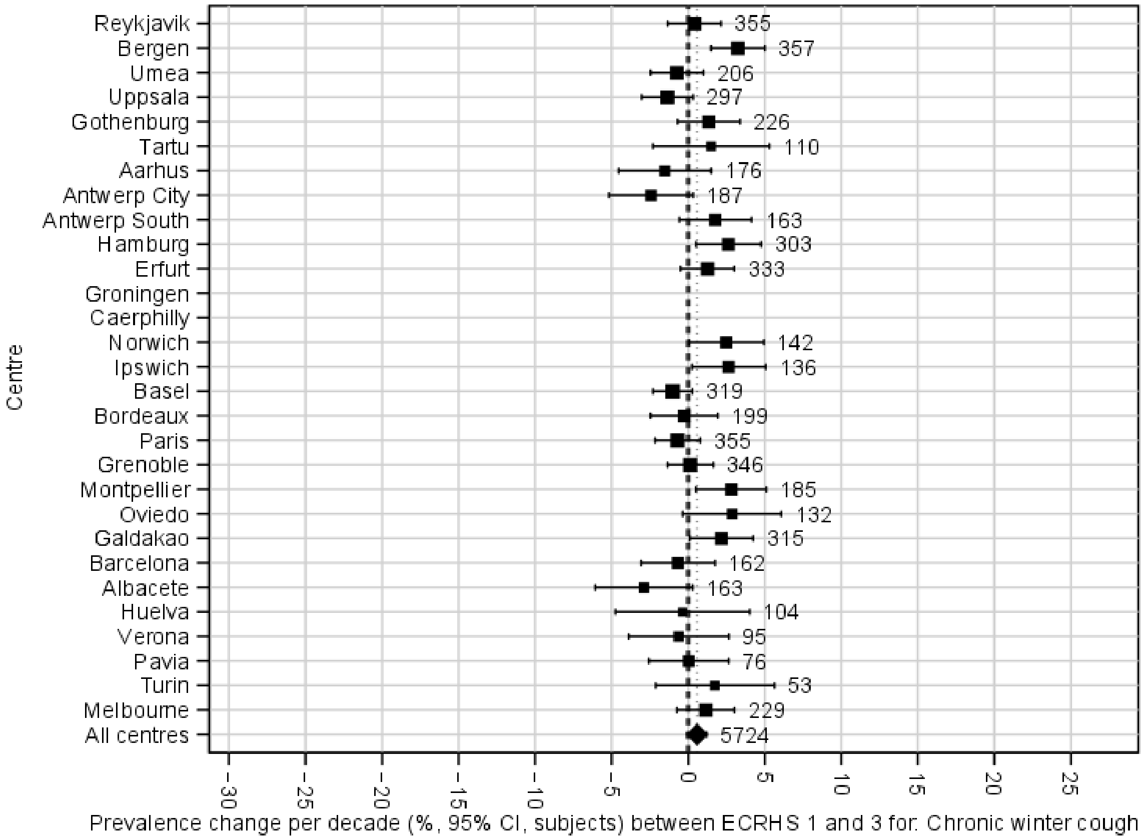

Figure E1(e). ECRHS III - ECRHS I change in prevalence (adjusted for non-response) estimated by generalised estimating equations in each centre for chronic winter phlegm

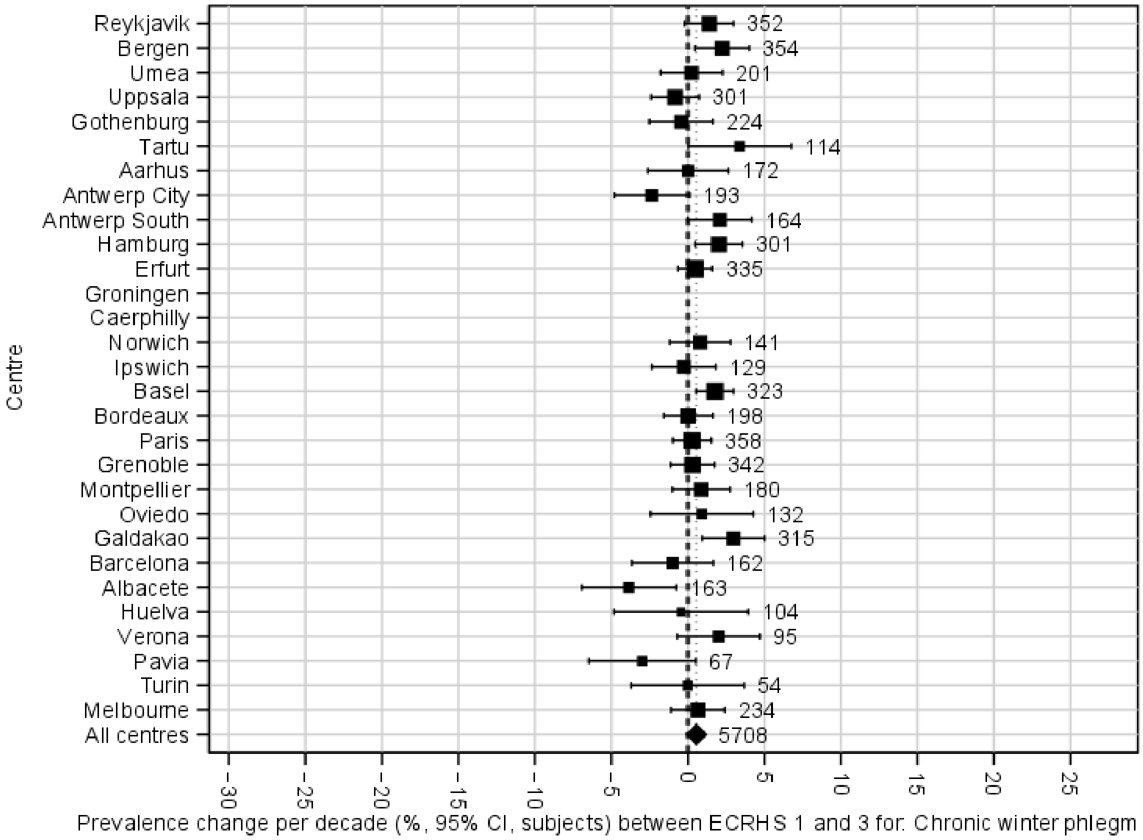

Supplement: Supplementary file 1 [file thoraxjnl-2016-209596supp001.pdf]
